# Serum exosomal miR‐16‐5p functions as a tumor inhibitor and a new biomarker for PD‐L1 inhibitor‐dependent immunotherapy in lung adenocarcinoma by regulating PD‐L1 expression

**DOI:** 10.1002/cam4.4638

**Published:** 2022-03-28

**Authors:** Hua‐Lin Chen, Yi‐Ping Luo, Mu‐Wen Lin, Xiao‐Xia Peng, Mei‐Liang Liu, Yong‐Cun Wang, Shu‐Jun Li, Dong‐Hong Yang, Zhi‐Xiong Yang

**Affiliations:** ^1^ Department of Pulmonary Oncology Affiliated Hospital of Guangdong Medical University Zhanjiang China; ^2^ Department of Oncology Affiliated Hospital of Guangdong Medical University Zhanjiang China

**Keywords:** biomarker for inhibitor‐dependent immunotherapy, LUAD, PD‐L1, serum exosomal miR‐16‐5p, tumor suppressor

## Abstract

**Objectives:**

We aimed at investigating whether serum exosomal miR‐16‐5p could be utilized as an immunotherapy biomarker in lung adenocarcinoma (LUAD) patients administered by programmed cell death ligand‐1 (PD‐L1) inhibitors, and to evaluate its functions in LUAD progression.

**Methods:**

Sixty LUAD sufferers and 20 healthy controls (HCs) were covered in this work. We applied both IHC and WB to examine PD‐L1 level in clinical tissue samples and utilized WB to quantify PD‐L1 expression in LUAD cells and LUAD xenograft tissues, respectively. Transmission electron microscopy (TEM), WB, and nanoparticle tracking analysis (NTA) were executed to confirm the exosomes isolated from serum specimens and cell culture media. To quantify of exosomal miR‐16‐5p level from serum and culture medium of cultured cell, qRT‐PCR experiment was utilized. The connection between tissue PD‐L1 level and serum exosomal miR‐16‐5p expression in PD‐L1‐positive sufferers administered by PD‐L1 inhibitors was verified using Spearman correlation coefficient analysis. In addition, the overall survival (OS) and progression‐free survival (PFS) rates among PD‐L1 inhibitor managed sufferers were acquired through a follow‐up visit. Finally, we used a group of assays, including 5‐bromo‐2′‐dexoyuridine (BrdU) and colony formation test, wound healing experiment, flow cytometry, and nude mice xenograft experiment, to explore the functions of circulating exosomal miR‐16‐5p on LUAD cell proliferation, apoptosis, and migration, as well as tumor development, respectively.

**Results:**

PD‐L1 expression was positively related to T stage (tumor size stage), and PD‐L1 inhibitor treatment reduced the PD‐L1 expression and mitigated T stage in PD‐L1‐positive LUAD sufferers. For PD‐L1‐positive LUAD sufferers, elevated PD‐L1 expression or reduced serum exosomal miR‐16‐5p level were linked to longer PFS and OS upon PD‐L1 inhibitor treatment. The number of exosomes in patient's serum was more than that in the serum of healthy individuals, and PD‐L1 inhibitor treatment decreased the number of serum‐derived exosomes in PD‐L1‐positive LUAD sufferers. Exosome‐derived miR‐16‐5p was downregulated in patient's serum and cell culture medium, and this was negatively linked to tumor stage and PD‐L1 expression. Meanwhile, PD‐L1 inhibitor treatment could increase the serum exosomal miR‐16‐5p expression, and the expression change of serum exosomal miR‐16‐5p was diametrically related to PD‐L1 after the treatment. Moreover, the overexpression of PD‐L1 accelerated tumor growth and decreased the exosomal miR‐16‐5p content in cell culture media, while exosomal miR‐16‐5p overexpression in cell culture media inhibited tumor development by decreasing the PD‐L1 expression. Exosomal miR‐16‐5p overexpression in cell culture media also depressed LUAD cell proliferation and migration, and stimulated cell apoptosis, especially in the cells which cultured in the mediums with PD‐L1 inhibitor in vitro.

**Conclusions:**

Serum exosomal miR‐16‐5p may be a latent tumor inhibitor and a new biomarker for PD‐L1 inhibitor‐dependent immunotherapy in LUAD by regulating the PD‐L1 expression.

## Graphical abstract

1.Exosomal miR‐16‐5p was downregulated in human LUAD serum samples and cell culture media. 2.Serum exosomal miR‐16‐5p was related to the tumor size stage and PD‐L1expression of LUAD patients. 3.Overexpression of exosomal miR‐16‐5p repressed cell proliferation and migration, stimulate cell apoptosis, and restrain tumor growth by lowering the PD‐L1 expression.

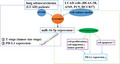

## 1 INTRODUCTION

According to the most recent epidemiological statistics, lung cancer (LC) keeps the leading causation of cancer‐associated death globally.^1^ Although the LC mortality is declining and the patient survival rate is rising, particularly for non‐small cell lung cancer (NSCLC) in USA,^2^, ^3^ LC morbidity and mortality appear a upward tendency in China in recent years.^3^ Histologically, NSCLC occupies 85% of LC, with lung adenocarcinoma (LUAD) being the most prevalent type.^4^ Moreover, LUAD makes up almost half of all kinds of LC, and its morbidity and mortality are rising in singulos annos, particularly among women and young adults.^5^, ^6^ Although substantial advances in LUAD diagnosis and conventional therapies such as surgery, radiotherapy, and chemotherapy, overall survival has not improved considerably, and the percentage of 5‐year survival is still under 20%.^6^ Increasing evidence has revealed that immunotherapy, benefits a variety of malignancies, particularly LUAD.^7^ Programmed cell death ligand‐1 (PD‐L1) (another name: CD274 or B7‐H1) expression indicates a suppressed immunity system, which allows tumor escape immune attack, causing tumors cells to grow and metastasize,^8^ and it is the sole FDA‐authorized biomarker for LUAD sufferers treated by immune checkpoint inhibitors (ICIs).^9^ There are long‐term beneficial responses in NSCLC patients after receiving ICI drugs that target on the PD‐L1;^10^, ^11^ however, only a fewer percentage of sufferers have a response to this immunotherapy.^12^ Thereby, biomarkers that are suitable for identifying the right type of patients are urgently needed in clinic.

Exosomes are biologically active small membrane vesicles (diameter: 30–150 nm) released from virtually all categories of cells, including cancer cells, and perform a variety of functions due to their rich and diverse components, such as cytosolic proteins, RNAs (mRNAs, microRNAs/miRNAs, circRNAs, lncRNAs), DNA, metabolites (lipids, cytokines), and else molecules.^13^, ^14^ Exosomes are discovered in multifarious body fluids, such as saliva, blood (whole blood, serum, and plasma), urine, cerebrospinal fluid, breast milk, bile, and malignant effusions.^15^, ^16^ Remarkably, exosomes have been utilized as possible biomarkers for cancer‐targeted therapies, including immunotherapy, in clinical trials due to their abundance, stability, polyfunctionality, and propensity to be detected in circulation.^17^, ^18^ It has been shown that the mechanism of ICI drug action is through blocking the interaction between the cell surface marker PD‐1 on T or B cell and its binding partner the PD‐L1 on the tumor cell, which subsequently causes the restoration of T cells (such as CD8^+^ T cell) proliferation then to resist the tumor‐induced immune inhibition.^19^ Interestingly, tumor‐derived exosomes (TEX) can inhibit CD8^+^ T cell proliferation on the one hand, and it can also lead to the dilation of CD4^+^ T cells on the other hand. However, normal cell‐released exosomes only function on the proliferation of all T cells.^20^ It is highly likely that exosomes involve in the ICI drug‐induced immunotherapy. Exosomes can also function as communication vehicles: their inclusions, safeguarded by a membrane from possible degeneration by extracellular enzymes during transportation, execute various biological functions in target cells.^21^ Exosomal miRNAs influence cell (including immune and cancer cells) biological process, such as maturation, proliferation, differentiation, and activation, by modulating targeted mRNA translation or stability.^22^, ^23^ It has been shown that miR‐16‐5p produced from M1 macrophage‐derived exosomes can boost T cell‐dependent immune response in gastric cancer tissues by targeting PD‐L1 expression,^24^ implying that exocrine‐derived miR‐16‐5p can contribute to immunotherapy for abnormal PD‐L1 expression in cancer. Sun and colleagues attested that CD274 (an alias of PD‐L1) was overexpressed in laryngeal squamous cell carcinoma (LSCC) tissues and the elevated level of CD274 was relative to a lower survival rate among LSCC patients. Based on information on databases, miRTarBase and StarBase v2.0, has‐miR‐16‐5p could negatively modulate the CD274 expression. Sun et al. further verified that miR‐16‐5p may function as an underlying biomarker for PD‐L1‐dependent immunotherapy.^25^ In a separated study, the authors illustrated that an elevated expression of miR‐16‐5p functions as a latent marker to demonstrate the effectiveness for melanoma sufferers under anti‐PD‐1 therapy.^26^ The above evidences indicate that miR‐16‐5p may directly or indirectly boost patient's immune system upon ICI‐induced immunotherapy. Furthermore, circ0021205 is overexpressed in the NSCLC tissues and cells. The upregulation of circ0021205 boosts the proliferation, migration, and invasion of human LUAD cells.^27^ It is worth to know that miR‐16‐5p upregulation partially reverses the effect of circ0021205 overexpression in the test using human LUAD cells.^27^ Together, it is clear that miR‐16‐5p exerts a repressive function in the malignant progression of LUAD; specifically exosomal miR‐16‐5p may refer to tumor immunity via regulating PD‐L1 expression. However, the expression of miR‐16‐5p in LUAD tissues and cells is unknown. In current work, we will evaluate the expression and role of circulating exosomal miR‐16‐5p in LUAD sufferers and cultured tumor cells and investigate whether serum exosomal miR‐16‐5p can be utilized as a biomarker to identify LUAD sufferers suitable for PD‐L1 inhibitor treatment.

## 2 MATERIALS AND METHODS

### 2.1 Patients and clinical specimens

**FIGURE 1:**
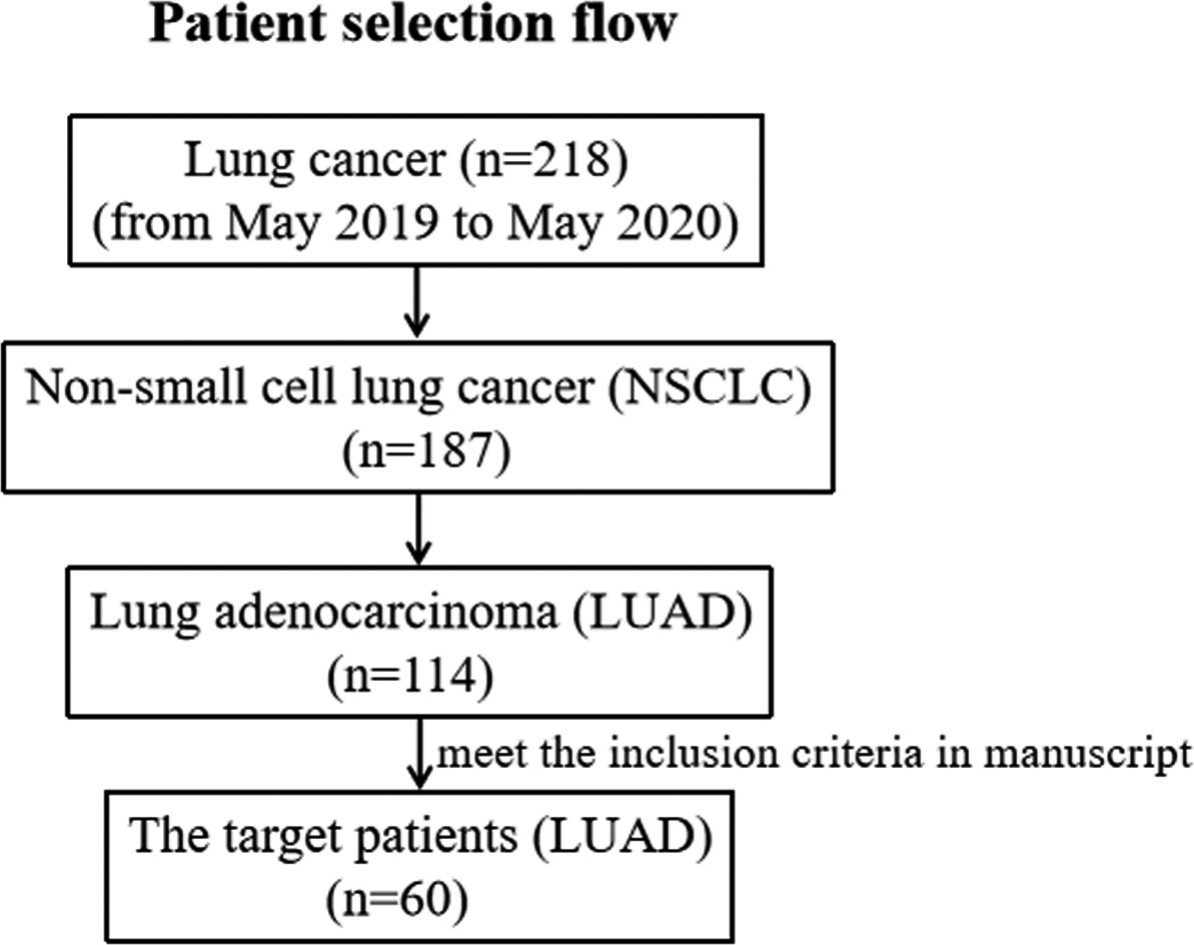
The flow chart depicting patient selection

The present work included 60 LUAD patients and 20 healthy volunteers who participated in body checks (as healthy controls [HCs]) in our hospital from May 2019 to May 2020. The patient selection is illustrated in a flow chart in Figure 1. From clinical instances of patients, basic clinical information such as gender, age, TNM (clinical/tumor progression stage), tumor location, smoking status, and Tstage (tumor size stage) were obtained. Patients who were found to have positive PD‐L1 expression were given a survival assessment and a performance status (PS) score based on the National Comprehensive Cancer Network (NCCN) guidelines, and those with a projected survival time >3 months and PS score ≦ 2 scores of two were given PD‐L1 inhibitors for 15 weeks. Moreover, after the accomplishment of PD‐L1 inhibitor‐based immunotherapy, these patients received a follow‐up visit to obtain information on PFS and OS. The following were the inclusion criteria: (1) All cases were initially diagnosed with LUAD in our hospital through histopathology of fiberoptic bronchoscopy; (2) No LUAD‐related surgery, radiotherapy, chemotherapy, immunotherapy, and other anticancer treatments were performed before admission; (3) No autoimmune diseases; (4) No blood‐related diseases; (5) No history of immunosuppressive drugs; (6) No other tumors; (7) No other fearful diseases; (8) Clinical case data were complete. Fresh LUAD tissues were obtained by fiberoptic bronchoscopy biopsy within 24 h of admission for immunohistochemical (IHC) staining (for each sample, tissue volume = 1 cm^3^) and western blotting (WB) (for each sample, quality = 20 μg) experiments to detect PD‐L1 expression, and 5 ml of peripheral venous blood samples were gathered on the day of admission for HCs population and all the LUAD patients, and on the days both before and after treatment with PD‐L1 inhibitors for LUAD patients who were treated by PD‐L1 inhibitors on an empty stomach at 7:30–9:30 AM for isolation and identification of exocrine of serum for detection of miR‐16‐5p content. The blood samples and fresh tissues were offhandedly kept in liquid nitrogen followed by refrigerated at −80°C. The current research protocol was authorized by the ethics committee of Guangdong Medical University's Affiliated Hospital and was carried out following the Helsinki Declaration's ethical principles. All recruited patients or their families provided written informed consent before the start of this trial.

### 2.2 IHC staining

Utilizing the PD‐L1 kit (22C3 pharmDX; Dako), IHC staining was executed to confirm the PD‐L1 protein level in fresh LUAD tissues by a fiberoptic bronchoscopy biopsy. Before IHC staining, the fresh LUAD tissues were frozen into 5‐μm‐thick tissue slices. The IHC staining method was carried out and reported utilizing prior literature as a guide.^28^ The final results were divided into three degrees by utilizing tumor proportion score (TPS) according to the proportion of tumor cells with membranous staining as reported by Miyazawa T et al.,^28^ which were negative staining (TPS <1%), weak positive staining (TPS = 1–49%), and strong positive staining (TPS ≥50%). PD‐L1‐positive staining was seen in all tumors with TPS ≥1%.

### 2.3 Cell line and cell culture

Shanghai Jianglin Biological Technology Co., Ltd. provided the BEAS‐2B cell (human normal bronchial epithelial cell line) and A549, PC9, and HCC827 cell (human LUAD cell lines). RPMI‐1640 media (Thermo Fisher Scientific), which included 10% FBS (Invitrogen), penicillin (100 U/ml), and streptomycin (100 mg/ml) (Gibco, BRL) were used for culturing the cells mentioned above. Then, a humidified incubator containing 5% CO2 was employed for incubating the cells at 37°C.

### 2.4 Cells transfection

Gene‐Pharma provided miR‐16‐5p mimic and negative control mimic (NC mimic). Gene‐Pharma created the recombinant plasmid of pcDNA3.1‐PD‐L1 and the corresponding empty pcDNA3.1 plasmid vector (NC vector). When a 70–80% of cell density was observed, serum‐free media was utilized to replace the original medium. For cell transfection, miR‐16‐5p mimic and NC mimic (100 nM), NC vector and pcDNA‐PD‐L1 (120 nM) were transfected into HCC827 or PC9 cells by utilizing Lipofectamine 3000 kits (Invitrogen). After 48 h, the mRNA expression of miR‐16‐5p in HCC827 or PC9 cells was evaluated using qRT‐PCR, while the protein expression of PD‐L1 was assessed using WB.

### 2.5 Exosome extraction

On the grounds of the manufacturer's guidelines, exosomes were isolated from human peripheral serum samples and cell culture media using the HIEFF™ Quick Exosome Isolation Reagent (41201ES50; Yeasen) (from serum) and the Total Exosome Isolation Kit (cell culture medium) (Cat#4478359; Invitrogen). The operation steps were described as reported in the previous study.^29^, ^30^

### 2.6 Transmission electron microscopy (TEM)

Referring to previously published research, TEM assays were performed at room temperature to identify the morphology of exosomes in serum and cell culture medium.^29^, ^31^ In summary, 20 mg of exosomes from serum and 10 μl of exosomes samples from cell culture medium were prepared and placed onto the carbon‐coated 300 (serum)/200 (cell culture media)‐mesh copper meshes (Beijing Zhongjing Keyi) for adsorption for 2 min (serum)/1 min (cell culture media). 2% phosphotungstic acid was applied to the meshes for counterstaining for 1 min. The samples were then dried with the filter papers. Exosomes were observed using a JEM‐1200 exii TEM (JEOL) at 80 kV (from serum)/120 kV (from cell culture media).

### 2.7 Nanoparticle tracking analysis (NTA)

Exosomal diameter size distribution in serum was evaluated utilizing NTA. Exosomes were deliquated in PBS buffer (1X; Biological Industries) at an initial concentration of 5 μg/ml, then 100–500‐fold (approximately 20–100 particles/frame) at pH 7.0. Exosomes were injected into sample chambers at room temperature, and exosomal size and concentration were assessed with NTA at 11 positions using Zeta View PMX 110 (Particle Metrix) and ZetaView 8.04.02 software which has been calibrated by 110‐nm polystyrene particles.

### 2.8 5‐bromo‐2′‐dexoyuridine (BrdU) proliferation assay

After the NC mimic or miR‐16‐5p mimic were transfected into HCC827/PC9 cells for 48 h, the cell culture media‐derived exosomes were gathered and utilized to treat HCC827/PC9 cells which cultured in the mediums with or without the drug (PD‐L1 inhibitor:10 μg/ml anti‐PD‐L1 antibody [LY3300054]), and the cells were named as NC mimic‐HCC827/PC9‐exosomes, miR‐16‐5p mimic‐HCC827/PC9‐exosomes, and miR‐16‐5p mimic‐HCC827/PC9‐exosomes+PD‐L1 inhibitor. The function of exosomal miR‐16‐5p from cell culture medium on HCC827/PC9 cell proliferation was determined using the 5‐bromo‐2′‐dexoyuridine (BrdU) assay. Importantly, 5 × 10^5^ NC mimic‐exosomes infected HCC827/PC9 cells, miR‐16‐5p mimic‐exosomes infected HCC827/PC9 cells, and miR‐16‐5p mimic‐HCC827/PC9‐exosomes + PD‐L1 inhibitor infected HCC827/PC9 cells were cultivated in 12‐well plates for BrdU assay. After the cells adhered to the wells, 10 μM of BrdU solution was dropped in each well for an additional 8 h of culture. The normal medium (10% FBA in DMEM) was used to replace the medium containing BrdU solution and cultured for additional 24 h. The cells were then harvested for immunostaining with BrdU (Abcam, RRID: ab142567, 1:500) and 4′, 6‐diamino‐2‐phenylindole (DAPI) (1 μg/ml; Invitrogen).

### 2.9 Colony formation assay

NC mimic‐exosomes, miR‐16‐5p mimic‐exosomes, and miR‐16‐5p mimic‐exosomes + PD‐L1 inhibitor infected HCC827/PC9 cells were cultured lasting 10 days in 6‐well plates with 1 × 10^3^ cells/well to form colonies. The cells were washed by 1 ml PBS/well and fixed for 15 min using 1 ml 4% paraformaldehyde (Beyotime Institute of Biotechnology) before being washed by 1 ml PBS/well and incubated for 15–20 min using 500 μl Giemsa stain (Sigma‐Aldrich; Merck KGaA). After rinsing away the excess staining solution by PBS, the colonies were photographed under an ×200 magnification of inverted light microscope (IX71; Olympus) and calculated with ImageJ software (version 4.0; National Institutes of Health).

### 2.10 Wound healing assay

In 24‐well plates, 1 × 10^5^ cells/well of NC mimic‐exosomes/miR‐16‐5p mimic‐exosomes/miR‐16‐5p mimic‐exosomes + PD‐L1 inhibitor infected HCC827/PC9 cells were seeded for wound healing assay for 24 h. When the cells in each well achieved 90% confluency of cell density, they were scraped by a 20‐μL sterile pipette tip. After washing injured monolayers of cells using PBS to discard detached cells and debris, each well was incubated with fresh media. At 0 and 24 h, the wounds in each well were photographed. The ImageJ software was used to calculate the cell migration rate (National Institutes of Health). At 0 h, the wound was deemed to have 100% of average migratory cells.

### 2.11 Flow cytometry for apoptosis assay

Next, 5 × 10^4^/500 μl medium of NC mimic‐exosomes/miR‐16‐5p mimic‐exosomes/miR‐16‐5p mimic‐exosomes + PD‐L1 inhibitor infected HCC827/PC9 cells were cultured into 24‐well plates lasting 24 h in CO2 incubator at 37°C, and then trypsinized, washed, and resuspended at 100‐μL binding buffer for flow cytometry. The cells were stained by Annexin‐V‐fluorescein isothiocyanate (VFITC) (5 μl, ab14085; Abcam) and propidium iodide (PI) (10 μl; BioLegend) in the dark for 15 min at room temperature. The rate of apoptotic cells were assessed using flow cytometry and the FlowJo software (v10.0.7; Becton, Dickinson, and Company) (Beckman Coulter).

### 2.12 Nude mice xenograft experiment

Thirty male BALB/C nude mice with 4–5 weeks old and 18.0–20.0 g of weight were offered by Vital River Laboratory Animal Technology and randomly divided into three groups (10 mice/group): NC vector, pcDNA‐PD‐L1, and pcDNA‐PD‐L1 + miR‐16‐5p mimic‐HCC827‐exosomes. All mice were used and raised obeying the Health Guide about the Care and Use of Laboratory Animals from National Institutes, and the research programme was confirmed by our hospital's Ethics Committee of Animal Research and executed conforming to the NIH Guidelines in the US. Then, 1 × 10^7^ of HCC827 cells transfected with NC vector or pcDNA‐PD‐L1, as well as 1 × 10^7^ of the HCC827 cells transfected with pcDNA‐PD‐L1 and subsequently treated with exosome extracted from HCC827 cells infected with miR‐16‐5p mimic, were inoculated subcutaneously into the right chelidon space. Tumor sizes on 7, 14, 21, 28, and 35 days were assessed to map a tumor growth curve. On day 35, after completing a tumor size assessment, all animals were anesthetized with 3% sevoflurane and sacrificed through cervical dislocation. The xenograft tumor tissues were photographed and weighted and kept at −80°C.

### 2.13 WB

WB was implemented to assess PD‐L1 level in LUAD tumor tissues (which were acquired from LUAD patients before and after a 15 weeks‐treatment with PD‐L1 inhibitors), HCC827 cells transfected with NC vector or pcDNA‐PD‐L1, and xenograft tumor tissues (which were obtained from different HCC827 cells (NC vector, pcDNA‐PD‐L1, pcDNA‐PD‐L1 + HCC827‐miR‐16‐5p mimic‐exosome). Moreover, WB was used to identify the expression of TSG101 and CD63 (exosome superficial marker proteins) in exosomes isolated from serum samples and cell culture medium. The WB procedure was carried out exactly as previously reported.^24^, ^32^ The primary antibodies utilized in this study were listed below: PD‐L1 (1:1000; Abcam), TSG101 (1:2000; Abcam), CD63 (1:1000; Proteintech), β‐actin (1:2000; Abcam), and Calnexin (1:2000; Cell Signaling Technology). β‐Actin was employed as an internal control of PD‐L1 for standardized quantification, while Calnexin was utilized as an internal reference of TSG101 and CD63

### 2.14 qRT‐PCR

According to the techniques described in the previous research, qRT‐PCR was carried out to assess the content of exosomal miR‐16‐5p in serum specimens and cell culture media, and the miR‐16‐5p level in cells ^33^. The 2^‐ΔΔCt^ method was adopted to quantify the relative expression of the miR‐16‐5p, and U6 was utilized as an internal control gene. The corresponding primer sequences displayed as follows:

miR‐16‐5p, F: 5’‐CTTAAGAACCCTCCTTACTC‐3′, R: 5’‐AAGCTACCCTAGGGGAAGGA‐3′. U6, F: 5’‐GCTTCGGCAGCACATATACTAAAAT‐3′, R: 5’‐CGCTTCAGAATTTGCGTGTCAT‐3′.

### 2.15 Spearman correlation coefficient analysis

The link between the expression change of PD‐L1 in LUAD tumor tissue and exosomal miR‐16‐5p in serum generated before and after therapy was examined using Spearman correlation coefficient analysis in LUAD sufferers with positive PD‐L1 expression who received PD‐L1 inhibitors.

### 2.16 Statistical analysis

The SPSS 21.0 software was adopted for statistical analysis, and the findings were displayed as mean ± standard deviation, number (*n*), and percentage/rate (%). The independent samples *t*‐test and Student's *t*‐test were performed for comparing the quantitative data between two groups. The X^2^ test**/**nonparametric Mann–Whitney *U*‐test**/**Fisher's exact test were ran to compare percentage/rate data between groups. Paired data were compared utilizing either the paired *t*‐test or the one‐sample *t*‐test. The survival curves of PFS and OS between the two groups were compared by employing a log‐rank test (Kaplan–Meier method). The correlations of quantitative data between two groups were examined with Spearman correlation coefficient analysis. *p* < 0.05 was deemed as statistically significant.

## 3 RESULTS

### 3.1 LUAD tissular PD‐L1 protein level was connected with the T stage/tumor size stage and the curative effect of PD‐L1 inhibitors

**FIGURE 2:**
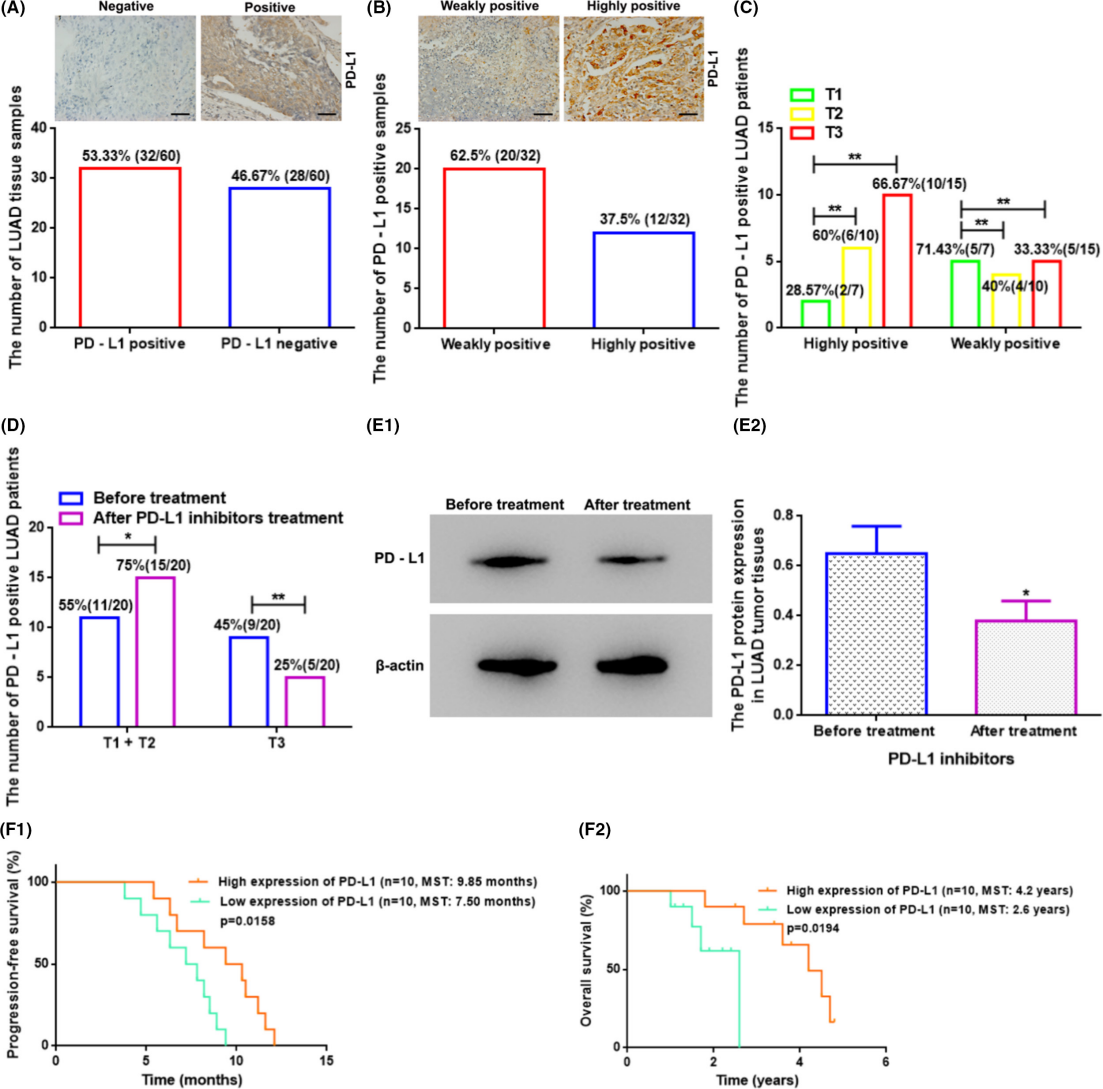
The PD‐L1 protein expression level in the LUAD tissues was associated with the T stage/tumor size stage and the therapeutic effect of PD‐L1 inhibitors treatment. (A) The number of PD‐L1‐positive and PD‐L1‐negative LUAD tissue samples detected by IHC. Superjacent figure: Representative images of IHC for PD‐L1‐positive and PD‐L1‐negative LUAD tissues. Scale bars: 50 μm. (B) The number of PD‐L1‐weakly positive and ‐highly positive LUAD tissue samples detected by IHC. Superjacent figure: Representative figures of IHC for PD‐L1‐weakly positive and ‐highly positive LUAD tissues. Scale bars: 50 μm. (C) The PD‐L1 level was related to the T stage in PD‐L1‐positive LUAD sufferers. T: primary tumor; T stage: tumor size stage; T1, T2, and T3 stage: tumor diameter size of primary tumor ≤3 cm, =3–5 cm, and >7 cm, respectively. ** vs. T1, *p* < 0.01. (D) The number of PD‐L1‐positive LUAD patients with different tumor size stages before and after treatment with PD‐L1 inhibitors. *p < 0.05; ** vs. before treatment, *p* < 0.01. (E) The PD‐L1 protein level in the LUAD tumor tissues before and after treatment with PD‐L1 inhibitors. * vs. before treatment, *p* < 0.05. (F) The relationship between PD‐L1 level and PFS/OS after PD‐L1 inhibitor treatment in PD‐L1‐positive LUAD sufferers. LUAD, lung adenocarcinoma; PD‐L1, programmed cell death ligand‐1; MST, median survival time

PD‐L1 protein level in fresh LUAD tissues derived from fiberoptic bronchoscopy biopsy was detected by IHC, and the positive result was shown as brown‐yellow granular staining of the cell membrane in tumor cells, and the results exhibited that 53.33% (32/60) of tissue samples presented PD‐L1 positive expression and 46.67% (28/60) of tissue samples showed no PD‐L1 expression (Figure 2A). Among PD‐L1 positive patients, 62.5% (20/32) showed weakly positive, whereas 37.5% (12/32) were strongly positive (Figure 2B). Moreover, there were no statistical differences in gender, age, TNM stage, tumor location, or smoking status between PD‐L1‐positive and ‐negative sufferers, or between PD‐L1‐highly positive and ‐weakly positive patients (*p* > 0.05) (Table 1), indicating the IHC results were comparable. Furthermore, in PD‐L1‐positive LUAD sufferers, the PD‐L1 level was connected with T stage (tumor size stage), specifically, when compared to patients at T1 stage (tumor size of primary tumor ≤3 cm) (*n* = 7), the patients at T2 or T3 stage (T2, tumor size **=** 3–5 cm, *n* = 10; T3, tumor size >7 cm, *n* = 15) showed an increased PD‐L1 highly positive rate and a decreased PD‐L1 weakly positive rate (*p* < 0.01) (Figure 2C). Meanwhile, among PD‐L1 positive patients, 20 sufferers received PD‐L1 inhibitor treatment for 15 weeks. After treatment, we found that the number of patients at T1 and T2 stages augmented (*p* < 0.05) and at T3 stage reduced (*p* < 0.01) (20% [4/20] of patients decreased from T3 stage to T2 stage) (Figure 2D). Meanwhile, the WB assay exhibited that the LUAD tissular PD‐L1 protein level was decreased after therapy than before (*p* < 0.05) (Figure 2E). Moreover, 20 LUAD patients upon the PD‐L1 inhibitor treatment were categorized into PD‐L1 high‐expression and low‐expression groups based on the cutoff value as per the median expression value of PD‐L1. At the end of the 15‐week treatment, the follow‐up evaluation displayed that the PD‐L1 high‐expressed sufferers illustrated with a better curative effect with longer PFS and OS than the PD‐L1 low‐expressed sufferers (*p* < 0.05) (Figure 2F).

**TABLE 1:** The relationships between the clinicopathological features of lung adenocarcinoma patients and PD‐L1 protein expression

Parameters	PD‐L1 positive (*n* = 32)	PD‐L1 negative (*n* = 28)	*p*‐value	PD‐L1 highly positive (*n* = 12)	PD‐L1 weakly positive (*n* = 20)	*p*‐value
Gender						
Male	22	20	>0.05	8	14	>0.05
Female	10	8		4	6	
Age						
≥55	20	17	>0.05	7	13	>0.05
<55	12	11		5	7	
TNM stage						
I	5	4	>0.05	2	3	>0.05
II	12	10		4	8	
III	15	14		6	9	
The location of tumors						
Left lung	15	13	>0.05	6	9	>0.05
Right lung	7	5		3	4	
Left and right lungs	10	10		3	7	
Smoking status						
Non‐smoker	18	16	>0.05	7	11	>0.05
Smoker	14	12		5	9	

Abbreviations: PD‐L1: programmed cell death ligand‐1; TNM: clinical/tumor progression stage.

### 3.2 Serum exosomal miR‐16‐5p was downregulated and related to the T stage, tissular PD‐L1 level, and the curative effectiveness of PD‐L1 inhibitors in the PD‐L1‐ positive sufferers

**FIGURE 3:**
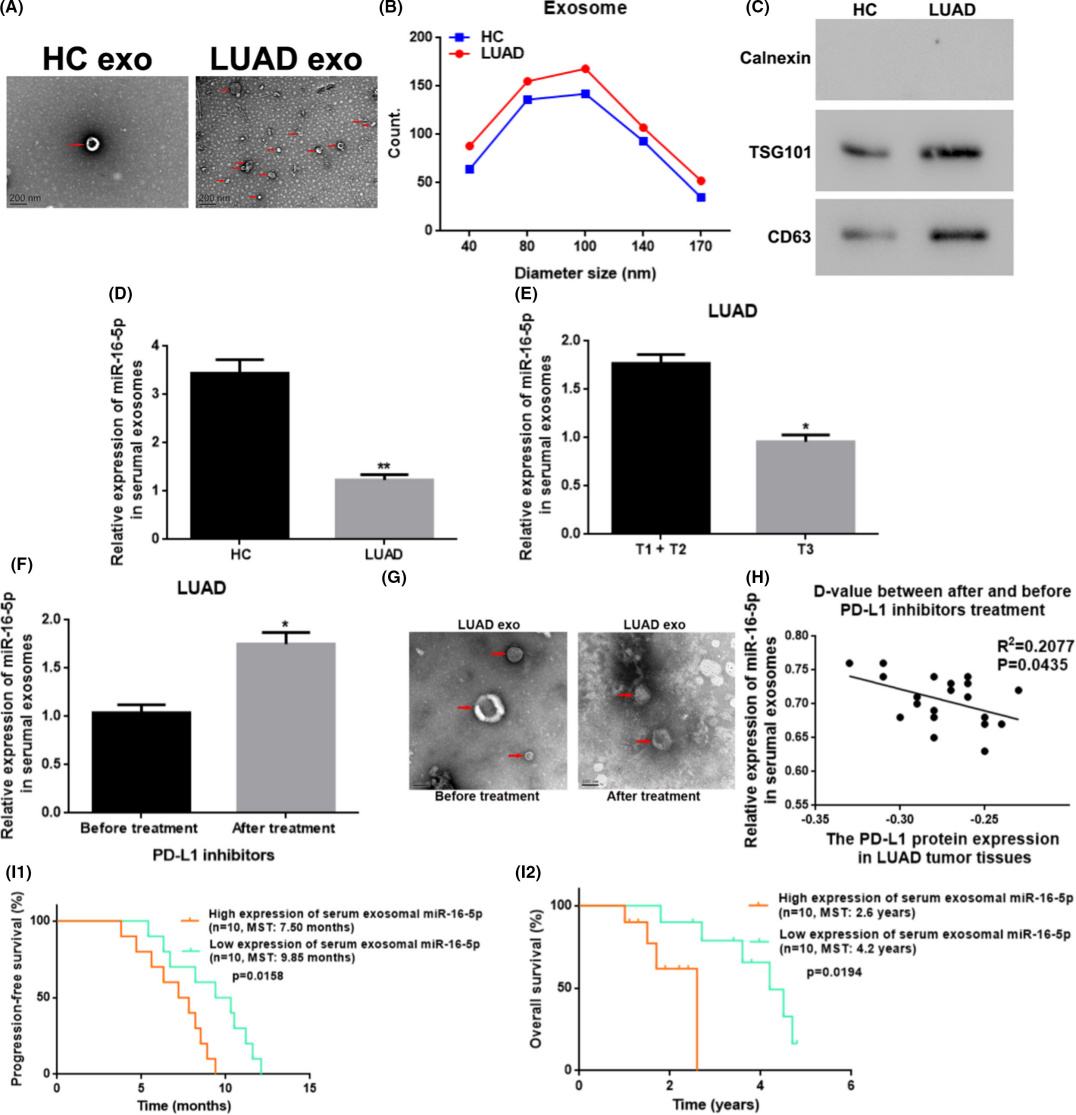
Serum exosomal miR‐16‐5p was downregulated and related to the T stage/tumor size stage, the PD‐L1 level in the tissues, and the curative effect of PD‐L1 inhibitor treatment of the LUAD patients. (A) Morphology of serum exosomes from healthy control people and LUAD patients under TEM. Scale bars: 200 nm**;** magnification: 30,000×; Red arrow: Point to the exosomes. (B) The diameter distribution of serumal exosomes from HC to LUAD patients using NTA. (C) The protein level of exosomal surface marker protein of TSG101 and CD63 in serum exosomes from HC to LUAD sufferers by WB. (D) The relative miR‐16‐5p level in serum exosomes from HC to LUAD sufferers with qRT‐PCR. ** vs. HC, *p* < 0.01. (E) The relative miR‐16‐5p level in serumal exosomes from LUAD patients with different tumor size stages. * vs. T1 + T2, *p* < 0.05. (F) The relative miR‐16‐5p expression in serumal exosomes from LUAD patients before and after treatment with PD‐L1 inhibitors. *vs. before treatment, *p* < 0.05. (G) Morphology of serum exosomes from PD‐L1‐positive LUAD patients before and after the treatment with PD‐L1 inhibitors under TEM. Scale bars: 100 nm; magnification: 30,000×. (H) The relationship between the expression changes of serum exosomal miR‐16‐5p and PD‐L1 protein in the LUAD tissues before and after treatment with PD‐L1 inhibitors. (I) The relation between serum exosomal miR‐16‐5p level and PFS/OS after PD‐L1 inhibitor treatment in LUAD sufferers with a positive PD‐L1 expression. HC, healthy control; LUAD, lung adenocarcinoma; exo, exosomes; T‐stage, tumor size stage; PD‐L1, programmed cell death ligand‐1; D‐value, difference‐value; MST, median survival time

We utilized a group of assays, including TEM, NTA, and WB, to characterize exosomes from serum samples. Exosomes in the patient's and HC's serum**s** all demonstrated as circular or circular‐like vesicles with intact membranes under TEM inspection, and more exosomes were observed in patient's samples compared with the samples from the control group (Figure 3A). The NTA assay revealed that the exosome sizes ranged from 40 to 170 nm, with an average size of 92 ± 8 nm. Particularly, the majority of exosomes showed a size distribution from 40 to 140 nm in diameter, and a peak size range of 80–100 nm was computed by employing the Image‐pro plus software (Figure 3B). Moreover, the serumal exosomes from both the patients and the control groups expressed CD63 and TSG101, but not Calnexin (Calnexin an integrin expressed on the endoplasmic reticulum but not in the exosomes). When compared to individuals from the control group, CD63 and TSG101 expressions were dramatically increased among LUAD sufferers (*p* < 0.01) (Figure 3C). As showed in Figure 3D, the serum exosomal miR‐16‐5p expressions were markedly decreased in LUAD sufferers (*n* = 60) when compared to those in HCs (*n* = 20) by qRT‐PCR assay (*p* < 0.01). Meanwhile, the serum exosomal miR‐16‐5p expressions were similarly declining in the LUAD sufferers with T1 tumor size stage (*n* = 13) compared to the patients with tumor sizes at T2 (*n* = 20) and T3 (*n* = 27) stages, respectively (*p* < 0.05) (Figure 3E). When the PD‐L1‐positive LUAD sufferers were treated with PD‐L1 inhibitors for 15 weeks, it was elaborated that the serum exosomal miR‐16‐5p expressions rose dramatically (*p* < 0.05) (Figure 3F) and the number of serum‐derived exosomes declined sharply (Figure 3G). Interestingly, after the 15‐weeks treatment with PD‐L1 inhibitors, the expression changes of serum exosomal miR‐16‐5p manifested a reverse correlation with PD‐L1 protein levels in tissues (*p* < 0.05) (Figure 3H). Meanwhile, the follow‐up data showed that the LUAD sufferers with a low serum exosomal miR‐16‐5p level displayed a better therapeutic effect with longer PFS and OS compared with the sufferers with a high serum exosomal miR‐16‐5p level (*p* < 0.05) (Figure 3I).

### 3.3 Exosome was successfully isolated from LUAD cell culture media, and the exosomal miR‐16‐5p was downregulated in LUAD cell culture media

**FIGURE 4:**
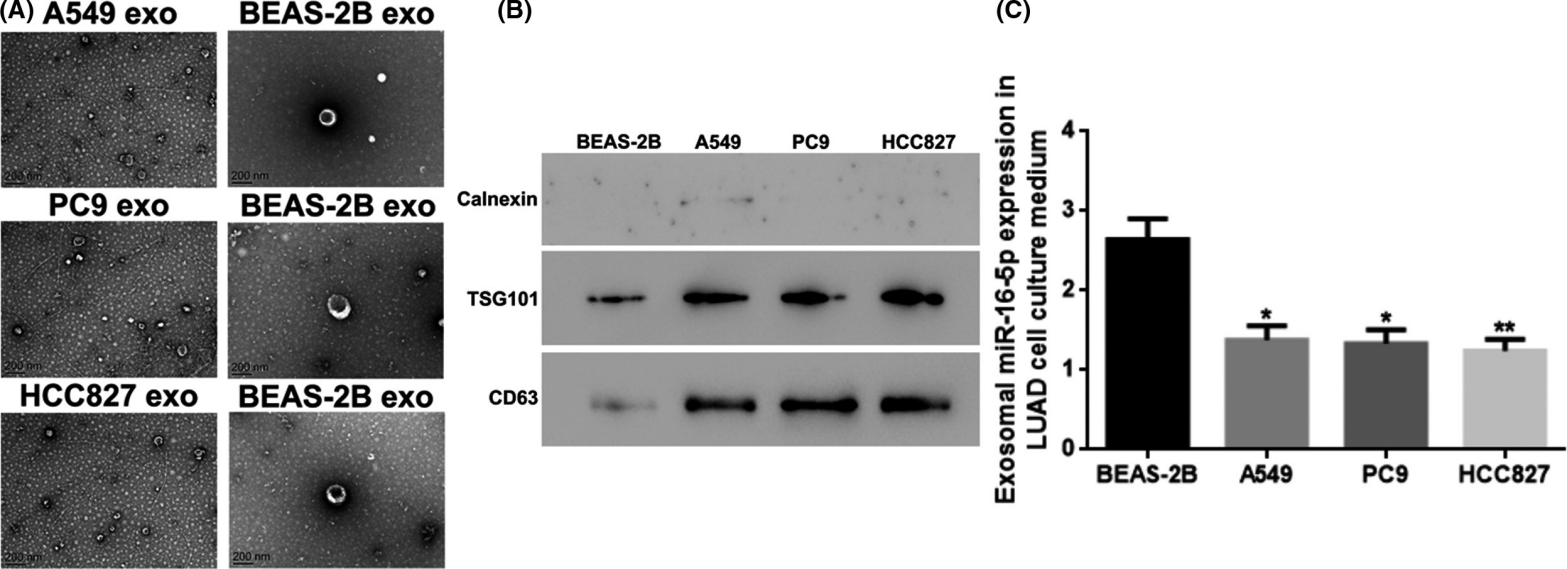
Exosomes were successfully isolated and identified out from LUAD cell culture media, and the exosomal miR‐16‐5p was lower expressed in the LUAD cell culture media. (A) The morphology of exosomes in cell culture media of A549, PC9, HCC827, and BEAS‐2B cell under TEM. Scale bars: 200 nm. (B) The protein level of exosomal surface marker protein of TSG101 and CD63 in exosomes in the cell culture media of A549, PC9, HCC827, and BEAS‐2B cell by WB. (C) The miR‐16‐5p expression in exosomes in the cell culture media of A549, PC9, HCC827, and BEAS‐2B cell with qRT‐PCR. *p < 0.05; ** vs. BEAS‐2B, *p* < 0.01. exo, exosomes; LUAD, lung adenocarcinoma

The exosomes from the media for A549, PC9, HCC827, and BEAS‐2B cells displayed typical round or round‐like vesicles with intact membranes under TEM, and more exosomes were observed in the media for A549/PC9/HCC827 cells than in the BEAS‐2B cell culture medium (Figure 4A). Meanwhile, the exosomal surface markers of TSG101 and CD63 were identified from the A549, PC9, HCC827, and BEAS‐2B cell culture media, and the expression levels of TSG101 and CD63 in the exosome from A549/PC9/HCC827 cell culture medium was higher than that from the BEAS‐2B cell culture medium (*p* < 0.05) (Figure 4B). Furthermore, the exosomal miR‐16‐5p levels in A549/PC9/HCC827 cell culture media were downregulated compared to those from BEAS‐2B cell culture medium (*p* < 0.05 or *p* < 0.01) (Figure 4C). The cell line HCC827 exhibited the lowest exosomal miR‐16‐5p level among tested cell lines, and thereby we selected this cell line for our subsequent study.

### 3.4 Upregulation of exosomal miR‐16‐5p restrained cell proliferation and migration, and stimulated cell apoptosis

**FIGURE 5:**
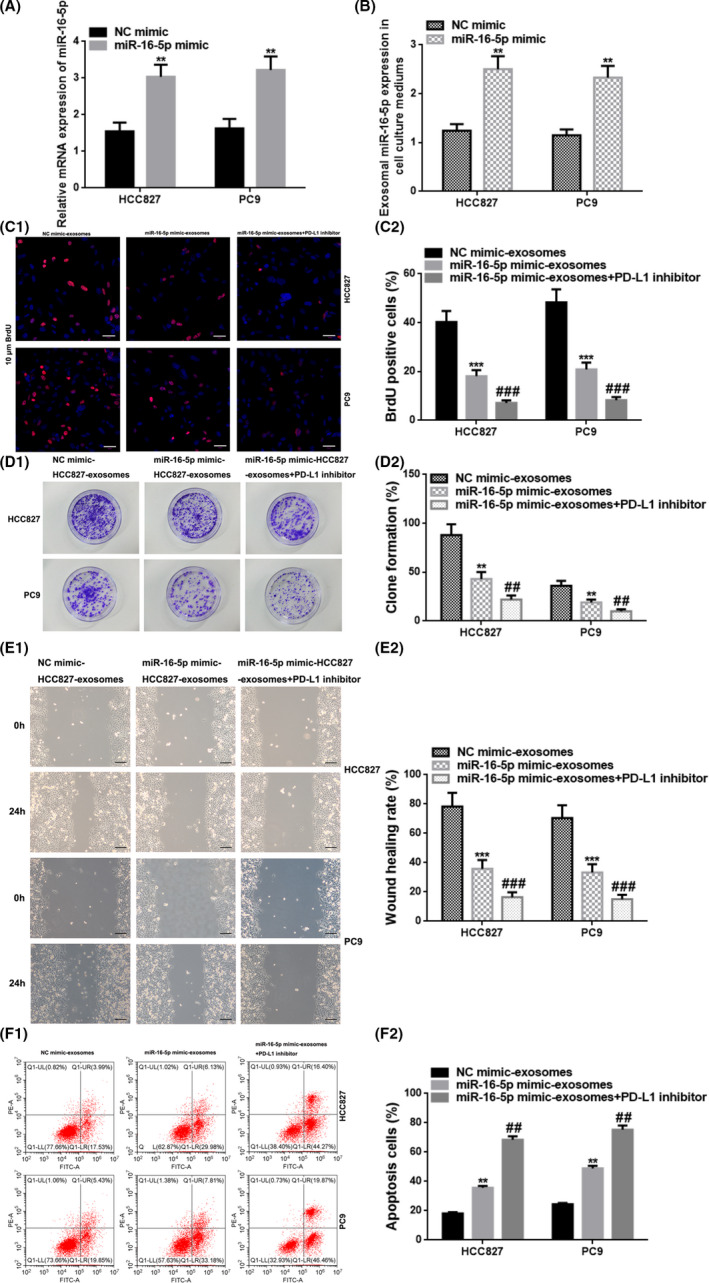
Upregulation of exosomal miR‐16‐5p in the cell culture medium restrained cell proliferation and migration, and accelerated cell apoptosis. (A) The relative miR‐16‐5p expression in HCC827 cells transfected into NC mimic or miR‐16‐5p mimic with qRT‐PCR. (B) The exosomal miR‐16‐5p level in the cell culture medium from HCC827 cells transfected into NC mimic or miR‐16‐5p mimic with qRT‐PCR. (C) Cell proliferation was estimated by BrdU immunostaining after treatment with exosomes from the HCC827/PC9 cell culture media transfected with NC mimic/miR‐16‐5p mimic/miR‐16‐5p mimic+PD‐L1 inhibitor. Red, HCC827/PC9 cells labeled with BrdU; blue, nuclei counterstained by BrdU. Scale bar, 50 μm. (D) The assessment of cell proliferation via colony formation assays after treatment. (E) Cell migration was determined with the wound healing assay at 0 and 24 h after treatment. Scale bar, 200 μm. (F) Cell apoptosis was assessed by flow cytometry after treatment. Q1‐UR: upper‐right side, early cell apoptosis. Q1‐LR: lower‐right side, late cell apoptosis. ** vs. NC mimic, *p* < 0.01; ** vs. NC mimic‐HCC827/PC9‐exosomes, *p* < 0.01; *** vs. NC mimic‐HCC827/PC9‐exosomes, *p* < 0.001; ## vs. miR‐16‐5p mimic‐ HCC827/PC9‐exosomes, *p* < 0.01; ### vs. miR‐16‐5p mimic‐HCC827/PC9‐exosomes, *p* < 0.001. NC, negative control; BrdU, 5‐bromo‐2′‐dexoyuridine; PD‐L1, programmed cell death ligand‐1

First, we successfully overexpressed miR‐16‐5p in HCC827/PC9 cells by transfecting miR‐16‐5p mimic and the transfection efficiencies were checked by qRT‐PCR (*p* < 0.01) (Figure 5A). Consequently, we discovered an upregulation of miR‐16‐5p in exosome in cell culture medium (*p* < 0.01) (Figure 5B). Next, the exosomes in cell culture media released from HCC827/PC9 cells which transfected into NC mimic or miR‐16‐5p mimic were collected. The HCC827/PC9 cells were named as NC mimic‐ HCC827/PC9‐exosomes or miR‐16‐5p mimic‐HCC827/PC9‐exosomes after treated by above collected exosomes. Moreover, the HCC827/PC9 cells which treated by cell culture media‐derived exosomes released from HCC827/PC9 cells which transfected with miR‐16‐5p mimic were also cultured in the cell culture medium containing PD‐L1 inhibitor and named as miR‐16‐5p mimic‐PC9‐exosomes+PD‐L1 inhibitor. Then, the HCC827/PC9 cell proliferation, migration, and apoptosis were determined and as shown in Figure 5C‐F, the miR‐16‐5p mimic‐HCC827/PC9‐exosome group had lower percentages of BrdU positive cells, colony formation, and migratory cells, and a higher proportion of apoptotic cells compared to those for the NC mimic‐HCC827/PC9‐exosome group (*p* < 0.01 or *p* < 0.001), and had higher percentages of BrdU positive cells, colony formation, and migratory cells, and a lower proportion of apoptotic cells when compared with those in the miR‐16‐5p mimic‐HCC827/PC9‐exosomes + PD‐L1 inhibitor group (*p* < 0.01).

### 3.5 Upregulation of exosomal miR‐16‐5p restrained the tumor development by inhibition of PD‐L1 expression

**FIGURE 6:**
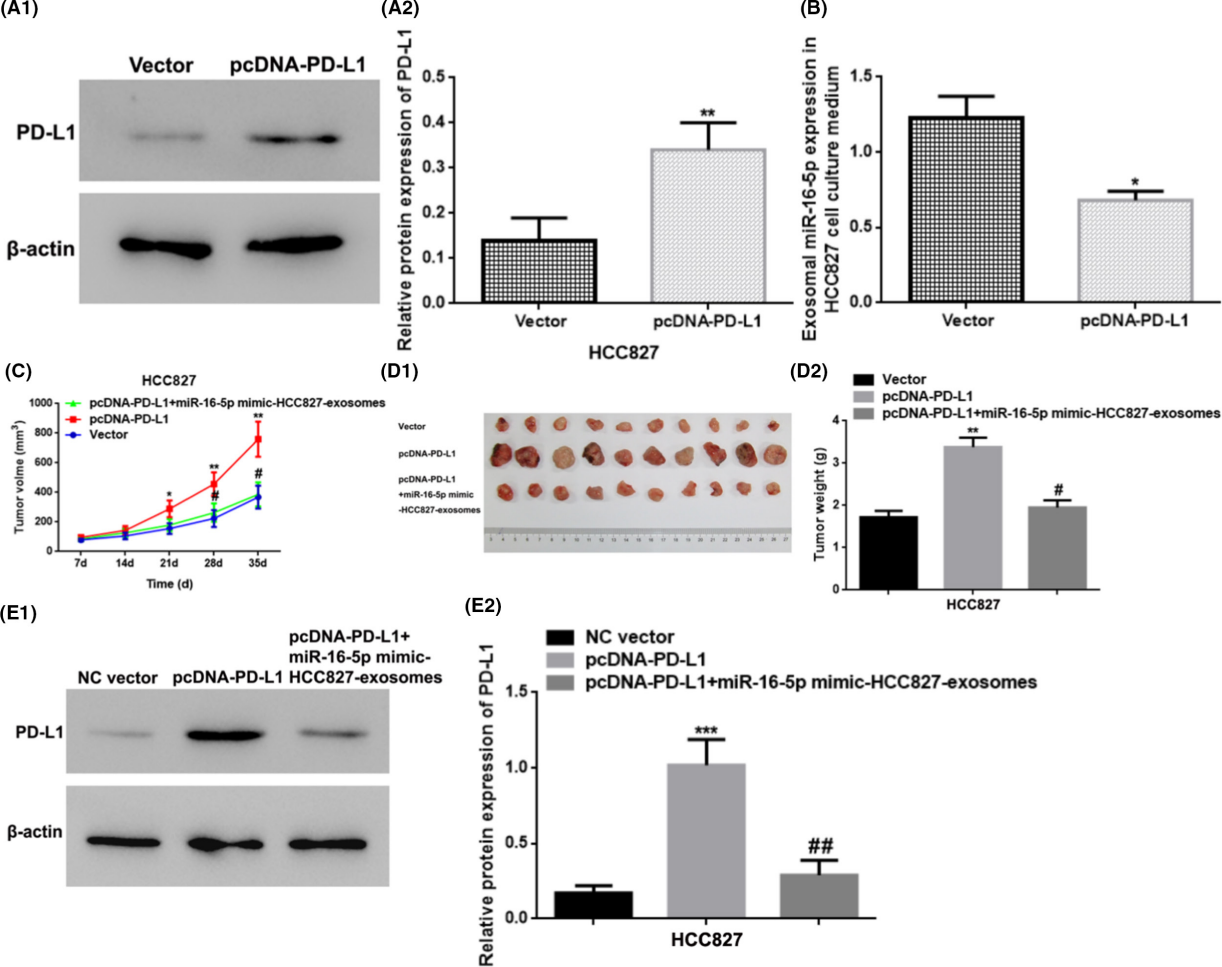
Upregulation of exosomal miR‐16‐5p in cell culture medium restrained the tumor growth by the suppression of the PD‐L1 level. (A) The relative PD‐L1 protein expression in the HCC827 cells by WB detection. ** vs. Vector (NC Vector), *p* < 0.01. (B) The exosomal miR‐16‐5p expression in the HCC827 cell culture medium by qRT‐PCR. * vs. Vector (NC Vector), *p* < 0.05. (C‐D) Tumor volume and weight of the xenograft tumor tissues in nude mice. * vs. Vector (NC Vector), *p* < 0.05; ** vs. Vector (NC Vector), *p* < 0.01; # vs. pcDNA‐PD‐L1, *p* < 0.05. (E) The relative PD‐L1 protein expression in xenograft tumor tissues from nude mice by WB. *** vs. NC Vector (Vector), *p* < 0.001; ## vs. pcDNA‐PD‐L1, *p* < 0.01. PD‐L1, programmed cell death ligand‐1; NC, negative control

We transfected HCC827 cells with a pcDNA‐PD‐L1 vector and the transfected cells showed PD‐L1 overexpression verified by WB (*p* < 0.01) (Figure 6A), and the overexpression then triggered a reduction in exosomal miR‐16‐5p in HCC827 cell culture medium (*p* < 0.05) (Figure 6B). To develop xenograft model, the HCC827 cells transfected into NC vector/pcDNA‐PD‐L1 vector/pcDNA‐PD‐L1 vector + exosome (extracted from cell culture media of miR‐16‐5p overexpressed HCC827cells) were inflooded into the subcutaneous area of right chelidon of nude mice. The findings indicated that the upregulation of PD‐L1 boosted the tumor growth which reflected by increased tumor volume and weight compared with the NC vector group (*p* < 0.05 or *p* < 0.01), and the overexpression of exosomal miR‐16‐5p in cell culture medium partially weakened the role of overexpression of PD‐L1 (*p* < 0.05) (Figure 6C, D). Finally, we detected the PD‐L1 protein level in tumor tissues of different xenografts using WB. The finds displayed that the PD‐L1 protein level was higher in the pcDNA‐PD‐L1 group than in the NC vector group; while it was increased in the pcDNA‐PD‐L1 group in a comparison to the pcDNA‐PD‐L1 + HCC827‐miR‐16‐5p mimic‐exosome group (*p* < 0.01 or *p* < 0.001) (Figure 6E).

## 4 DISCUSSION

In present work, we mainly highlighted that PD‐L1 upregulation enhanced the LUAD T stage **(**tumor size stage**)** and tumor development, which could be alleviated by using a PD‐L1 inhibitor. Furthermore, exosome‐derived miR‐16‐5p released from serum and cell culture medium in LUAD were downregulated, which was linked to T stage, LUAD tumor growth, and PD‐L1 level. Both the tissular PD‐L1 and serum exosomal miR‐16‐5p expressions were in connection with the therapeutic effects of PD‐L1 inhibitor in treating PD‐L1‐positive LUAD sufferers. The exosomal miR‐16‐5p generated from cultured LUAD cells may weaken cell proliferation and migration, accelerate cell apoptosis, and slow tumor development via decreasing the PD‐L1 expression. PD‐L1, a 40‐kDa transmembrane protein located on the cellular membrane, can regulate the activity of T‐cell receptor then to participate in T cell‐mediated immune reactions, is widely expressed in nearly all types of tumor cells and assists tumor to elude immune attack, making it useful as an immune checkpoint in clinical trials.^8^, ^24^ PD‐L1 is also a key ligand of PD‐1, which is found on the surfaces of enabled T cells. When PD‐L1 is coupled with PD‐1, it can suppress T cell response by causing apoptosis and slowing cell cycle, as well as promote immune escape, leading to tumor growth.^8^, ^34^ As a result, developing immunotherapy based on ICIs such as monoclonal antibodies combining the PD‐1/PD‐L1 pathway, subjected to extensive research in recent years, with promising results in treating various malignancies including LUAD.^35^, ^36^ PD‐L1‐positive expression in LUAD tissue has been considered as an important but a limited predictive biomarker for ICIs treatment in clinical trials due to only a small number of patients benefiting from taking ICIs/PD‐L1 inhibitors by displaying an remarkable effect of significantly prolonging survival period.^9^, ^37^ In our study, PD‐L1 level was identified positively related to the T stage (tumor size stage**)** in the PD‐L1‐positive sufferers, and that the treatment by PD‐L1 inhibitors could alleviate the grade of T stage by downregulating PD‐L1 level. The PD‐L1‐positive sufferers with higher PD‐L1 expression exhibited better responses with longer PFS and OS after a 15‐week of treatment with PD‐L1 inhibitors, indicating that the PD‐L1 expression may be a marker for predicting the potential therapeutic effect. The finding has a great potential to guild the clinical application of PD‐L1 inhibitor‐dependent immunotherapy in PD‐L1‐positive LUAD sufferers. Moreover, in a nude mice xenograft experiment, we discovered that PD‐L1 overexpressionstimulated tumor development. Complementally, in this work, the T stage was classified based on the tumor size, and the tumor growth was evaluated according to the tumor volume and weight, which may indirectly represent the tumor size**.** A meta‐analysis involving 11,444 patients and 47 studies revealed that an elevated PD‐L1 level was closely connected with poor prognosis (decreased OS) in NSCLC including LUAD. But, some individual characteristics such as gender and smoking status could affect PD‐L1 level, and PD‐L1 expression was also related to tumour size, and TNM stage.^38^ Therefore, to ensure comparability between groups, we analyzed clinical basic information data and found that no statistical differences between PD‐L1‐positive and ‐negative sufferers or between PD‐L1‐highly positive and ‐weakly positive sufferers on gender, age, TNM stage, tumor location, and smoking status. These results ensured the group comparability. Furthermore, PD‐L1 expression in tumors (including LUAD) detected by using the immunohistochemistry assay has been considered as an unneglectable step in the standard practice for immunotherapy with antibodies against PD‐1/PD‐L1, and blocking of the mutual action of PD‐1 with PD‐L1 can restrain tumor growth and possibly result in complete remission of cancer.^39^ In our work, we also utilized immunohistochemistry to identify PD‐L1 level in tumor tissues of 60 LUAD patients. We identified 32 cases with PD‐L1 expression, of which 12 cases showed strong‐positive PD‐L1 expressions and the remaining 20 cases exhibited weak positive PD‐L1expressions. The LUAD tumor tissues with positive PD‐L1 expressions were then used for further studies, and our findings supported the hypothesis that PD‐L1 promotes tumor development.

Almost all cell kinds, including cancer cell, generate exosomes and subsequently release to the peripheral blood, and exosomes contain a wide range of bioactive molecules (including miRNA), allowing them to be absorbed by other cells and perform stable activities.^13^, ^14^, ^15^, ^16^ Significantly, circulating exosomal miRNAs are more stable than other types of miRNAs and have been utilized as specific biomarkers to identify certain cancers.^40^, ^41^, ^42^, ^43^ While miRNAs often adversely affect the synthesis of target proteins via a distinctively combining with the 3′‐untranslated regions (3′‐UTRs) of the gene transcript, which subsequently triggering gene‐silencing.^44^ Exosomal miRNA‐16‐5p has been shown to specifically combine with the 3′‐UTRs of the PD‐L1 transcript in gastric cancer, therefore enhancing T cell‐based immune reaction.^24^ In current study, the exosomal miR‐16‐5p level in the serum of LUAD sufferers was downregulated, and this serum exosomal miR‐16‐5p expression was discovered in negative connection with tumor size stage and the PD‐L1 level in LUAD. Moreover, PD‐L1‐positive LUAD patients with a lower serum exosomal miR‐16‐5p expression demonstrated a better therapeutic effect with longer PFS and OS after upon a treatment with PD‐L1 inhibitors. Hence, serum exosomal miR‐16‐5p level may be a potential and newfangled immunotherapy biomarker for predicting the efficacy of PD‐L1 inhibitor.

We also conducted both the in vitro and in vivo studies to explore the unknown roles of serum exosomal miR‐16‐5p in LUAD. First, we isolated and characterized exosomes from patient's serum and cell culture medium. We discovered that exosomes were small membrane vesicles with the diameter range of 30–150 nm ^13^ and exosomes expressedspecific maker proteins TSG101 and CD63 but not Calnexin.^45^ In addition, we observed that cancer cells generate more number of exosomes than normal cells from the same organ.^46^ After PD‐L1 inhibitor treatment, the number of exosomes was reduced in serum in PD‐L1‐positive LUAD sufferers, this indicated that there was a relevance between exosomes and immunotherapy. Next, our findings illustrated that exosomal miR‐16‐5p was less abundant in the culture medium of LUAD cells (A549, PC9, and HCC827). Consequently, the HCC827 and PC9 cells were selected for subsequent studies because of its relative lower exosomal miR‐16‐5p content among the three cell lines. The exosomal miR‐16‐5p overexpression in HCC827/PC9 cell culture medium significantly restrained cell proliferation and migration, stimulated cell apoptosis, and restricted tumor development associated with the decreased PD‐L1 expression. The overexpression of miR‐16‐5p inhibited human LUAD A549 and PC9 cell proliferation, migration, and invasion, according to Yang Y et al.^27^ Furthermore, circulating exosome‐associated miR‐16‐5p was downregulated in multiple myeloma (MM) sufferers who were resistant to the treatment with bortezomib (Bz) ,^47^ and serum exosomal miR‐16‐5p might be utilized as one of the possible multiple biomarkers panel for identifying esophageal adenocarcinoma.^48^ MiR‐16‐5p was also identified low expressed in hepatocellular carcinoma tissues and cells ,^49^ in chordoma tissue ,^33^ and in neuroblastoma patient‐derived xenografts with high MYCN expression.^50^ The upregulation of miR‐16‐5p depressed cell proliferation, migration, invasion, and metastasis, and tumor formation.^33^, ^49^, ^50^, ^51^ The online data from the database of Starbase forecasted that miR‐16‐5p and PD‐L1 can bind to each other, and the hypothesis was supported by the result of the dual‐luciferase reporter assay.^22^ Tao et al. also predicted and attested that miR‐16 targets human and mouse PD‐L1 by using bioinformatics analysis and luciferase reporter assay, respectively. The prostate cancer sufferers with high expression of miR‐16 exhibited positive association with biochemical recurrence‐free survival. Upregulation of miR‐16 expression blocked PD‐L1 expression, which lead to an activation of T cell, thus facilitating the radiotherapy in prostate cancer.^52^ Sera exosomal miR‐16 expression was significantly downregulated in esophageal adenocarcinoma (EAC) PD‐L1(+) patients compared with in EAC PD‐L1(−) patients.^53^ With reference to these evidences, we mainly adopted an indirect verification in this work, and we discovered that overexpression of PD‐L1 caused a decline in exosomal miR‐16‐5p expression in HCC827 cell culture medium and overexpression of exosomal miR‐16‐5p level caused a decrease of PD‐L1 protein level. Besides, the expression change of serum exosomal miR‐16‐5p was negatively in connection with PD‐L1 level in PD‐L1‐positve sufferers after treatment with PD‐L1 inhibitors. Together with the finds in vitro and in vivo assays, we speculated that serum exosomal miR‐16‐5p may function as a tumor inhibitor in LUAD by modulating PD‐L1 expression. Nevertheless, further investigations with a larger sample size are warranted to validate our findings.

Summarily, the findings from this study have a great potential in cancer clinic. For instance, although the PD‐L1 expression is the exclusive biomarker approved by FDA for applying ICIs for LUAD population and long‐lasting beneficial responses have appeared in the PD‐L1‐positive NSCLC sufferers treated with PD‐L1 inhibitors, only a few percentiles of patients have been benefited from this immunotherapy. Moreover, whether a patient can take the PD‐L1 inhibitor or not depends on the tumor tissue PD‐L1 expression, which requires an invasive way to acquire, by utilizing an IHC assay. Meanwhile, the tumor size, PFS, and OS are the key indicators for tumor progression or prognosis in clinic. In this study, the serum exosomal miR‐16‐5p expression was detected to be lower expressed in LUAD sufferers, in association with the tumor size. On the other hand, for the PD‐L1‐positive LUAD patients treated with PD‐L1 inhibitors, serum exosomal miR‐16‐5p content was declined compared with that before treatment; the change in the serum exosomal miR‐16‐5p level was negatively relevant to the tissular PD‐L1 expression. Moreover, the PD‐L1‐positive LUAD sufferers with higher PD‐L1 expression/lower serum exosomal miR‐16‐5p level showcased a favorable response after the PD‐L1 inhibitor treatment with longer PFS and OS. The findings in this study elaborated that serum exosomal miR‐16‐5p may be a new marker for predicting which LUAD sufferers are candidates for the PD‐L1 expression‐based immunotherapy. In addition, serum exosomal miR‐16‐5p is more stable than serumal miR‐16‐5p, and the relevant samples can be obtained noninvasively. In vitro assay, we discovered the exosomal miR‐16‐5p expression in LUAD cell culture medium was also downregulated. In vivo experiments, we demonstrated that the upregulation of exosomal miR‐16‐5p played tumor‐suppressive role by downregulating the PD‐L1 expression. Additionally, the relation between the serum exosomal miR‐16‐5p and the tumor size/PD‐L1 expression in LUAD patients suggest that the former one may be a tumor inhibitor in LUAD progression via regulating the PD‐L1 expression. Our study sheds a new light on developing therapeutic agents for LUAD patients by targeting on the expression of serum exosomal miR‐16‐5p.

## 5 CONCLUSIONS

The finds in this research mainly reflected that the expression of tissular PD‐L1/serum exosomal miR‐16‐5p had something to do with Tstage (tumor size stage) and the therapeutic effect of PD‐L1 inhibitors in PD‐L1‐positive LUAD sufferers. After treatment, the expression change of serum exosomal miR‐16‐5p was diametrically in connection with that of PD‐L1. Exosomal miR‐16‐5p level was lower expressed in the patient's serums and LUAD cell culture media. The restoration of exosomal miR‐16‐5p expression visibly depressed cell proliferation and migration, and facilitated cell apoptosis, as well as restrained tumor growth by decreasing the PD‐L1 expression. So, serum exosomal miR‐16‐5p may be utilized as a tumor inhibitor and a feasible biomarker for PD‐L1 inhibitor‐dependent immunotherapy in LUAD via regulating PD‐L1 expression.

## CONFLICT OF INTEREST

The authors asserted no potential conflict of interest in regard to the study, authorship, and/or publication of present manuscript.

## AUTHOR CONTRIBUTIONS

Yang ZX and Yang DH designed research, edited, and revised manuscript; Chen HL drafted the manuscript; Luo YP analyzed data and prepared figures; Lin MW, Peng XX, Liu ML, Wang YC, and Li MJ performed experiments, interpreted results of experiments, and agreed the ultimate version of this article.

## ETHICS APPROVAL

The clinical research program was permitted by the ethics committee of our hospital and complied with the Code of Ethics of the World Medical Association (Declaration of Helsinki), printed in the British Medical Journal (18 July 1964). We obtained written informed consent from the sufferers/family before study initiation. For animal experiments, all mice were raised on the strength of the Health Guide about the Care and Use of Laboratory Animals from National Institutes. The investigation program was authorized by the Ethics Committee of Animal Research of our Hospital and performed on the basis of the guidelines laid down by the NIH in the US. In order to minimize pain or discomfort, all animals were anesthetized with 3% sevoflurane before they were sacrificed by cervical dislocation.

## Data Availability

All data produced or analyzed involved in present research are enrolled in this published paper.
